# Diverse fungi associated with partial irregular heartwood of *Dalbergia odorifera*

**DOI:** 10.1038/srep08464

**Published:** 2015-02-16

**Authors:** Sisheng Sun, Xu Zeng, Dawei Zhang, Shunxing Guo

**Affiliations:** 1 Institute of Medicinal Plant Development, Chinese Academy of Medical Sciences & Peking Union Medical College, Beijing, 100193, China; 2 College of Food and Biological engineering, Xuchang University, Xuchang, 461000, China

## Abstract

*Dalbergia odorifera* T. Chen is a medium-sized evergreen tree that produces purple-brown heartwood called JiangXiang in traditional Chinese medicine, the formation process of which takes several decades. In this study, a standard culture method was used to isolate fungi from the wounded and normal stems of *D. odorifera* aiming to investigate the difference between the two types of wood. To characterize the spatial colonisation of endophytic fungi, an anatomical study was undertaken using the two different types of wood of *D. odorifera*. A total of 320 wood segments were placed on PDA plates and 87 fungal isolates were obtained. Only two fungi were isolated from the healthy white wood tissue, whereas 85 fungi were found in the purple-brown wounded-wood tissues. The two isolates from 160 white healthy wood tissues were assigned to *Bionectriaceae* sp., and the rest in wounded wood tissues were analyzed to 12 fungal species, indicating both a high fungal diversity and colonization rate in the purple-brown wounded wood. There was a difference in fungal species composition between coloured and white wood samples collected from the same tree. *Eutypa* sp. was the most commonly isolated species in the purple-brown wounded wood.

D*albergia odorifera* T. Chen is a medium-sized evergreen tree belonging to the Leguminosae family. It was originally distributed in Hainan, China and was gradually introduced and cultivated in Guangdong, Fujian, Zhejiang, Guangxi, and Yunnan, China^1^. The heartwood of *D. odorifera*, named as “JiangXiang” in traditional Chinese medicine (TCM), has been included in Chinese Pharmacopoeia for decades and is widely used for dissipating stasis, stopping bleeding, regulating “Qi”, and relieving pain^2^. In Korea, the heartwood is also used for the treatment of blood stagnation syndrome, ischemia, swelling, necrosis and rheumatic pain^3^. Volatile oil and flavonoids are the major medicinal components in *D. odorifera*. The former has a variety of pharmacological effects, including anticoagulant, anti-myocardial ischemia, anti-platelet, and antimicrobial activitie^4^,^5^, whereas the latter also exhibits many functions, including anti-inflammatory, anticoagulant, antitumor, vasodilative, antioxidant, and antihyperlipidemic activities^6^,^7^,^8^,^9^,^10^.

*D. odorifera* tree has been widely employed not only as a kind of medicinal material in the pharmaceutical industry, but also famous for luxury furniture and crafts, owing to sweet fragrance, beautiful surface, high density, and economic value of the heartwood. However, wild *D. odorifera* trees have become endangered as a result of overexploitation in the past years and artificially cultivated plants still cannot meet the short-term demand for medicine usage. Therefore, the protection of wild resources of the plant is an urgent task, and the development of new methods for rapid induction of heartwood with great medicinal value is needed.

Under natural conditions, *D. odorifera* trees occasionally suffer from environmental stresses and biotic stresses including lightning, typhoon, or other mechanical damage, and thus are wounded. After a period, the wound will bleed purple resin. Thereafter, the colour of the wounded wood will change from white to purple-brown and ultimately the irregular heartwood will be formed with attractive flavor and high medicinal values. The mechanism governing this process remains unclear at present. Taken the valuable agarwood as an example, researches have showed that fungi are beneficial for the formation of agarwood in Aquilaria malaccensis^11^. In flowering plants, 300,000 species are colonised by pathogenic fungi^12^. It is likely that the formation of *D. odorifera* heartwood induced by wounding might be similar to the formation of agarwood induced by fungi invasion.

The evolution of fungal phytopathogens toward a high degree of specialisation for individual plant species may be reflected in the different levels of specialization observed in extant plant-fungal interactions^12^. The first level may be seen in opportunistic parasites, which enter plants through wound or require otherwise weakened plants for colonisation. These fungal species are usually characterised by a broad host range but a relatively low virulence. These traits are similar to those of endophytic fungi of plants. The most commonly used definition of endophytic fungi is that of fungi inhabiting plant organs at some time in their life that can colonise internal tissues of plants without causing apparent harm to the host.

Heartwood, defined as the central layers of wood tree containing nonliving cells and nonfunctioning xylem tissue, is blocked with tannins, resins and oils. It is usually darker and more durable than the outer sapwood. Heartwood is different from the normally dead wood in the center of all tree trunks that provides structure and support for the tree but does not participate in the transport of sugars or water^13^,^14^,^15^,^16^.

The role of endophytic fungi in *D. odorifera* is still unclear. In the present study, wood samples from healthy and wounded *D. odorifera* were collected from a plantation in Puning, northeastern Guangdong province, China. Following wounding, wood colours changed from white to purple-brown after a period of time. When ignited, the purple-brown wood produced a distinct fragrance, which was unique to heartwood of. *D. odorifera*. We isolated and identified endophytic fungi in the wounded and normal uninjured wood of the trees using a standard culture method and characterized their relationships and differences. To determine the spatial distribution of endophytic fungi, we performed an anatomical study in an attempt to study the diversity of fungal species harboring in wounded wood of *D. odorifera* that shows evidence of heartwood presence. These fungi are candidate to be responsible for heartwood formation in *D. odorifera*.

## Results

### Fungal isolation and identification

**Figure 1:**
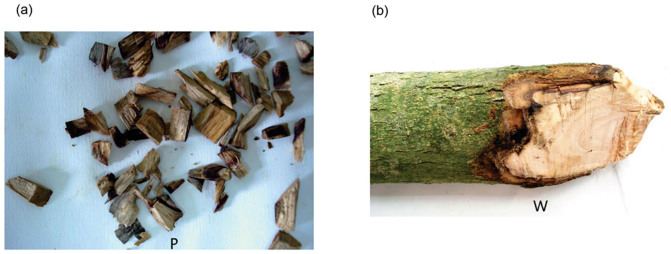
Two types of wood samples collected from stems of A *Dalbergia odorifera* T. Chen. P: purple-brown wounded wood indicating the presence of heartwood. (a), W: white healthy wood (b). The two samples were further sectioned based on colour characteristics and subjected to conventional fungal isolation procedures. The photographs of the wood in figures 1 were obtained by myself from Puning City, Guangdong Province.

Purple-brown wood segments were collected from nine wounded *D. odorifera* trees and white healthy wood stems were collected from three healthy plants growing naturally in an artificially cultivated condition. All trees were approximately seven years old. Small pieces of purple-brown wood were tested positive by ignition, which is similar to heartwood, but negative for white healthy wood. They had a diameter of more than 8 cm at breast height and did not form natural purple-brown heartwood. Fig. 1a shows samples of purple or purple-brown wounded wood of *D. odorifera*. Fig. 1b shows the healthy white wood, displaying the natural wood colour of the seven-year-old trees. A total of 320 wood segments were placed on PDA plates and 87 fungal isolates were obtained. Only two fungi were isolated from a 160 white healthy wood tissues, which were affiliated to Bionectriaceae sp.. On the contrary, 85 fungi were identified from purple or purple-brown wounded wood tissues, and belonged to 12 species (Table 1). These results illustrated the diversity of endophytic fungi in purple-brown wounded wood.

### Molecular identification and phylogenetic analysis

**Figure 2:**
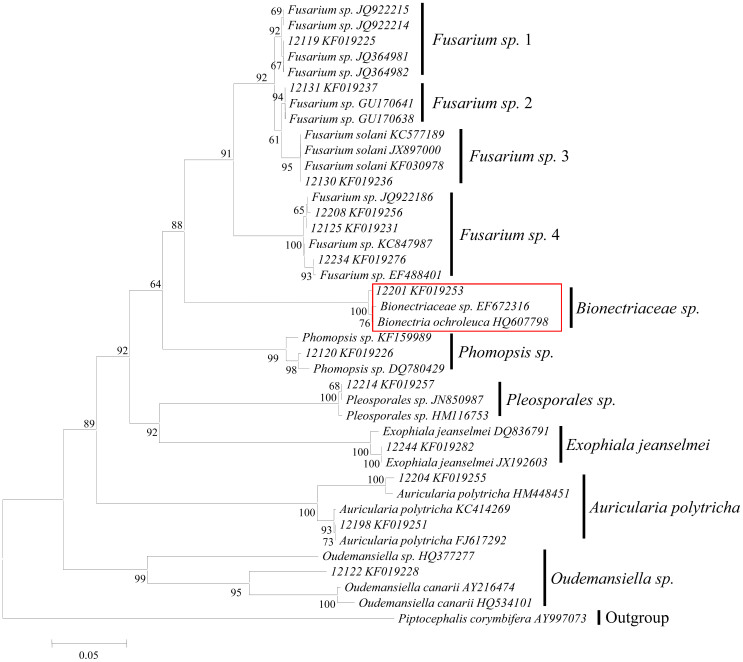
Phylogenetic relations among 10 isolates of endophytic fungi obtained in culture from healthy and wounded wood of *D. odorifera* and 8 representatives of the Ascomycota. Bootstrap numbers greater than 50% are indicated. A NJ tree was generated with MEGA software from the alignment of ITS sequences. Sequences from the reference taxa have GenBank accession numbers listed in parentheses. Bootstrap values (≥ 50%) are indicated with corresponding nodes. The sequence marked in red is the only one isolated from white healthy wood. *Piptocephalis corymbifera* was used as the outgroup. Bar indicates the nucleotide substitutions per site.

The obtained sequences were subjected to BLASTn analysis to reference sequences in GenBank. Phylogenetic tree showed that the isolates formed seven distinct clades (Fig. 2) with a majority of the bootstrap values well above 90%, reflecting an accurate phylogeny. In the neighbour-joining tree, three isolates 12119, 12130 and 12131 were closely related by a 92% bootstrap value, clustered strongly with the reference sequences in GenBank. For example, the ITS sequence from the cultured isolate 12120 from wounded wood was identified as *Phomopsis sp.*, wand was clustered by 98% bootstrap support with *Phomopsis* sp. (accession DQ780429). Isolate 12201 originated from white healthy wood, formed a strongly supported clade with *Bionectriaceae* sp. (accession EF672316). *Bionectria ochroleuca* (accession HQ607798) clustered by 64% bootstrap unsupport with 12120 that isolated from purple-brown wounded wood, which was identified to be *Phomopsis* sp.. *Fusarium* sp. (12208) and *Fusarium* sp. (12234) isolated from the wounded wood, clustered by 100% bootstrap support with both *Fusarium* sp. (accession JQ922186), and 12125, which was also isolated from purple-brown wounded wood. These were assigned as the same species. The sequence from the culture identified as *Auricularia polytricha* (12204) isolated from purple-brown wounded wood, clustered with 100% bootstrap support with *A. polytricha* (accession HM448451). The sequence from the culture identified as *A. polytricha* (12198) isolated from wounded wood, clustered with 100% bootstrap support with *A. polytricha* (accession HM414269), so that 12198 and 12204 were assigned as the same species (Fig. 2). This result indicates that isolate 12201 cultured from white healthy wood did differ from the fungi isolated from the purple-brown wounded wood of *D. odorifera* trees. There were different fungi in white and coloured woods, implying that there would be some correlations between fragrance and fungal species that could be discerned. This is one aspect of the results that could be further explored.

### Isolation frequency of endophytes

Isolation frequency was used for fungal diversity analysis. All cultured strains were subjected to sequencing. And the overall analyses revealed twelve fungal species in the purple-brown wounded wood and one in the healthy white wood. Among the isolated fungi, two belonged to Basidiomycota and the rest belonged to Ascomycota. Although *Eutypa* and *Eutypella* are closely related, the paucity reports designating them as two genera^17^,^18^,^19^, thus prevented us from identifying the species. Combining morphological and molecular identification, three fungi of 12123, 12126 and 12200 were identified as *Eutypa* sp., *Eutypella* sp. 1 and *Eutypella* sp. 2. Their colonies showed different characters and *Eutypa* sp. (12123) was the most frequent. Four species belonged to the genus *Fusarium*. The *Eutypella* genus comprised two species. The *Eutypa*, *Phomopsis*, *Oudemansiella* and *Pleosporales* genera comprised only one species each in purple-brown wounded wood. *Bionectriceae* genera comprised only one species in healthy white wood. Additional species were *Auricularia polytricha* and *Exophiala jeanselmei* (Table 2).

The overall colonisation frequency was determined as 53.125% for purple-brown wood segments, but only 1.25% for white healthy wood segments (Table 1). In the purple-brown wounded wood tissues, the dominant fungi were *Eutypa*, *Phomopsis*, *Oudemansiella* and *Fusarium*. The most frequently isolated species was *Eutypa* sp. in wounded wood (Table 2). The results showed that purple-brown wounded wood had not only a high fungal colonisation rate, but also a rich diversity of fungi. There was a difference in fungal species composition between coloured and white wood collected from the same tree.

### Anatomical identification and natural heartwood

**Figure 3:**
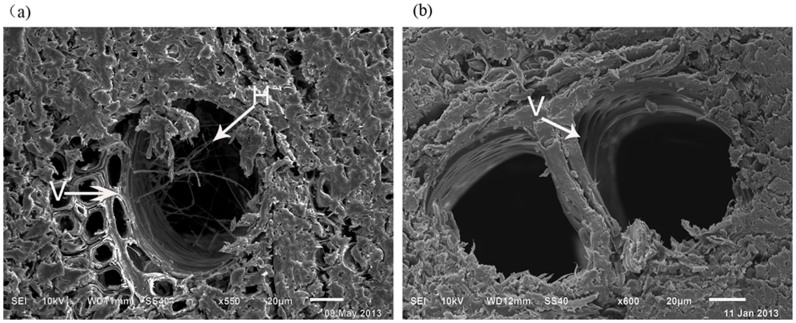
Scanning electron microscopy (SEM) images of the two types of wood of *D. odorifera.* Transverse section of purple or purple-brown wounded wood. Fungal hyphae in purple or purple-brown wounded wood were detected. The hyphae in tissue are indicated by arrows, with arrow 2 indicating that fungi hyphae occurred in a vessel of purple-brown wounded wood (a). Transverse section of white healthy wood. There were no hyphae in white healthy wood. Arrow 1 indicates the vessel present in the region (b). V: Vessel; H: Hyphae.

After anatomical identification, as showed in Fig. 3, some fungal hyphae were present in the vessels of purple-brown wounded wood. However, no hyphae was found in the vessel of healthy white wood. Overall, a total of 100 healthy white wood tissues were analyzed and no hyphae existed within the tissue surface or vessels.

**Figure 4:**
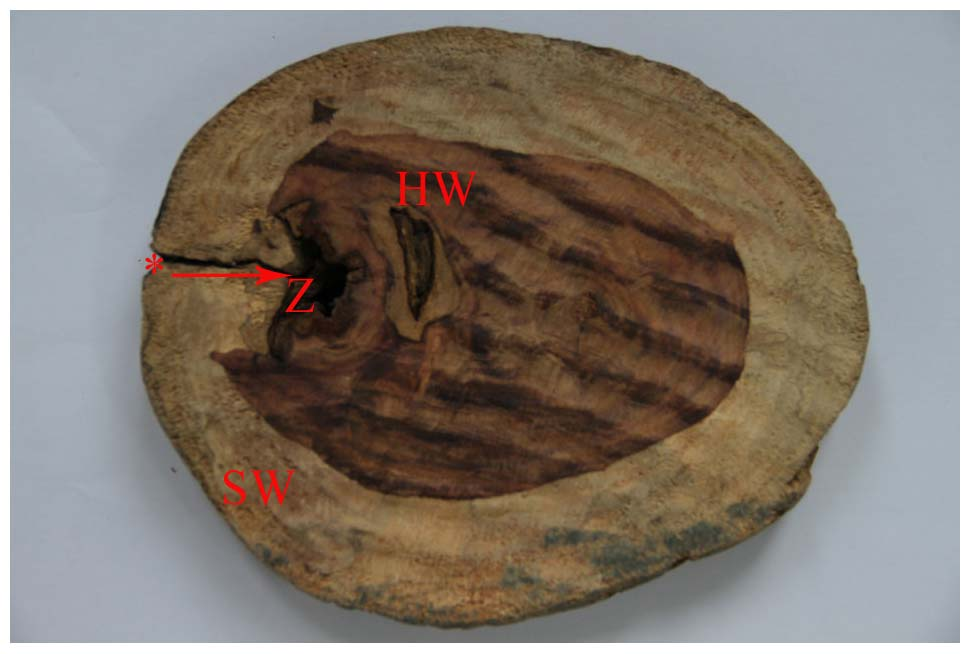
An example of a transverse section of a *D. odorifera* tree that has formed natural heartwood. It shows the wound site (_*_) and the infected site by fungi, sapwood and heartwood. Arrow represents the invasion of fungi from the wound site to the interior of the wood. SW: Sapwood; Z: Zone; _*_: Wound site; HW : Heartwood. The photographs of the wood in figures 4 was obtained by myself from from Zhaoqing City, Guangdong Province.

The fragrant resin was produced in some wood plant species when it was subjected to external injury, which was a defense response to plant living^20^,^21^,^22^. *D. odorifera* tree timber formed natural heartwood (Fig. 4) was provided by Mr. Zhu for the experiment, from a mountain in Gaoming District, Foshan City, Guangdong Province. It had died and formed wild natural heartwood. It was estimated to be 18 years old. The diameter of this timber was about 16 cm, the heartwood was 9 cm and the wounds size was about 4 cm in depth. From the appearance, we determined that it was wounded wood. A purple-brown heartwood had begun to form near the wound and a purple-brown substance seemed to surround the wound, suggesting that this substance have formed in response to microbial invasion and served to inhibit wound expansion. Inside the wounded area there was a zone, which provided conductive conditions for fungal growth and preserved adequate moisture for fungal growth under the conditions of high temperature and humidity in the southern China.

## Discussion

All plants in natural ecosystems appear to be symbiotic with fungal endophytes^23^. The endophytic fungi widespread in plants have been well studied, as they are found in ferns, lichens, algae, mosses, and vascular plants^24^,^25^,^26^. This study showed that the purple-brown wounded wood of *D. odorifera* was colonised by various fungi, whereas white healthy wood was colonised by only one endophytic fungus. The 85 fungi isolated from 160 purple or purple-brown wounded wood tissues can be identified to species, genus, or family level, including *Eutypa*, *Phomopsis*, *Oudemansiella*, *Fusarium*, *Eutypella*, *Pleosporales*, *Exophiala polytricha* and *Albonectria rigidiuscula*. *Phomopsis*, *Oudemansiella* and *Fusarium* fungi were the most frequent within the hosts, consistent with the findings of other studies^27^,^28^,^29^. *Eutypa*, the most frequently isolated genus, of which pathogens often infect grapevine trunks^19^, has the ability to turn white wood into desirable purple-brown wood. Two isolates from 160 white healthy wood tissues were assigned to the same species, *Bionectriaceae* sp.. The fungal diversity in purple or purple-brown wounded wood was greater than that in white healthy wood. They may be manipulated to induce heartwood production in plantation-grown trees. Further studies should test these isolates for induction of heartwood formation.

To obtain nutrients within the host cell, fungi must first breach the natural barriers of healthy plants. These barriers include chemical (including inhibitory compounds or the absence of stimulatory compounds needed for fungi development) and physical (cuticle, bark, cell wall, stomatal aperture or lenticel) elements. Some fungi lack the ability to penetrate these natural barriers, so that wound sites become the channel for their invasion. In the present study, the wounded wood of *D. odorifera* contained diverse fungi species, but the healthy white wood had only one species. This finding suggests that wounding and fungi play an important role in heartwood formation. In *Aquilaria malaccensis*, however, there was no difference in fungal species composition between the coloured and white wood collected from the same wounded sites^30^. This may be due to the fact that fungi can readily colonise the low-density wood of *A. malaccensis*. In contrast, the outside bark of *D. odorifera* is 1 cm thick and the wood is solid and dense, with sapwood harder than the wood of other trees.

Plant defenses basically appear as the formation of constitutive antimicrobial secondary compounds or induced antifungal compounds. The former occurring in healthy plants is likely to represent chemical barriers to infection and protect plants against pathogen attack. The latter absent in healthy plants, are synthesized in response to fungal attack or stress as part of the plant defense response and is restricted to the tissue colonised by fungus and the cells surrounding the infection site^31^. In this study, the formation of purple-brown wounded wood belonged to the latter category. There are many flavonoids in the hearwood of *D. odorifera*^32^. Flavonoids play an important role in resisting the invasion of pathogens for plant growth, development, flowering and fruit. It is estimated that 2% of the carbon fixed by photosynthesis is converted to flavonoids or other closely related compounds, and the majority of tannins are also converted to flavonoids^33^. Flavonoids are considered to be the active principles of many medicinal plants with health-related properties based especially on their antioxidant activities. The production of flavonoids may be induced by fungal invasion. Such results implied that the purple-brown substance formation of *D. odorifera* tree protects plant growth from external factors.

Heartwood formation in general is associated with the breakdown of the water transport system and the decrease in vitality of parenchyma tissue, radial transport processes (assimilate and ion movement), decrease in various physiological functions, and decomposition of soluble carbohydrates, by which storage substances are degraded or transformed into heartwood substances^15^,^16^. The process of red heart formation is assumed to develop in successive phases, leading to a cloudy appearance of several formation zones in cross section. As shown in Fig. 4, the formation of heartwood with *D. odorifera* is consistent with this recognition.

Plants are associated with microorganisms, including bacteria, actinomycetes and fungi, which are part of the natural ecosystem of healthy plants and occur in the main habitats of the leaf surfaces, rhizosphere and internal tissues. For this reason, studying the mechanisms of plant defense is of great importance to human well-being, as well as of great academic interest in evolutionary biology. Endophytic fungi have been found in most vascular plants including many medicinal plants, and have been isolated from diverse plant tissues including bark, leaves, seeds, petioles, fruits, wood and spines^25^,^34^. Our observations of injured *D. odorifera* trees with purple substances showed no apparent disease in the wild. They may represent endophyte traits resulting from invasion and colonization by external fungi. However, to date, the endophytic fungal complexes in adamant heartwood have not been reported. Whether the extremely hard texture of heartwood accounts for this scarcity of research on endophytic fungi isolated from this species is an open question.

Given the abundance and diversity of endophytic fungi, the formation of the purple-brown wounded wood may be due to the combined actions of a variety of fungi. Only a part of the *D. odorifera* tree is able to form this purple-brown wood*.* We inoculated *Eutypa* sp. into the sapwood of *D. odorifera* tree and found its role in inducing the sapwood discoloration. The discolored sapwood contains more flavonoids than whiter wood (following report). We hypothesized that purple-brown wood formation would be induced by a defense response to fungal invasion at wounded sites. The relationship between the formation of partial irregular heartwood and the invasion of fungi into *D. odorifera* trees need more artificial inoculation for further study. This study may be useful to those involved in traditional Chinese medicine as well as mycologists studying host-associated fungi. Whether the heartwood formation of *D. odorifera* is induced by the invasion of fungi awaits further verification and the underlying molecular mechanisms require further study.

## Methods

### Sample collection

Seven-year-old healthy and wounded timbers of *D. odorifera* were collected from Guangdong province (latitude 23°19′3.60″N and longitude 115°59′27.66″E), China. Diameter of the seven-year-old trees is about 7.5 cm, and they do not form regular heartwood in the innermost stem parts. The wounded timbers of *D. odorifera* were wounded about 2 years, and the wounds size was about 3 cm in depth. The plants were identified by Prof. Shunxing Guo, Institute of Medicinal Plant Development, Chinese Academy of Medical Sciences & Peking Union Medical College, China. Purple or purple-brown wood segments were ignited to check the heartwood fragrance. The plant materials were brought to the laboratory in sterile polyethylene bags and stored temporarily in a refrigerator. Wood segments were collected and placed in sterile microcentrifuge tubes. Endophytic fungi were isolated within 24 hours.

### Isolation of endophytic fungi

To eliminate epiphytic microorganisms, the two types of wood were subjected to surface-sterilization. Each sample was thoroughly washed under running tap water and then sterilized with 75% ethanol for 1 min followed by 3% NaClO for 8 min and 75% ethanol for 30 s, and rinsed three times with sterile distilled water and the pieces were blot-dried with sterile blotting paper. The samples were cut into 5 mm-thick segments. Approximately 4–5 segments were placed into potato dextrose agar (PDA) containing 50 μg/mL chloramphenicol and 50 μg/mL penicillin. Samples were incubated at 25°C in the dark. The fungi were observed every 2 days for more than 4 weeks^35^,^36^,^37^,^38^. The fungi from the segments were periodically isolated and identified according to morphological characteristics and then the hyphal tips were transferred to fresh PDA plates for purification and identification. We did not observe any additional fungi emerge after two months. Pure endophytic fungal strains were photographed and preserved in the laboratory of Mycology, Biotechnology Center, Institute of Medicinal Plant Development (IMPLAD), Chinese Academy of Medical Sciences (CAMS).

### Morphology-based identification

After isolation, cultures were identified at the genus or species level by culture and microscopic characters. Identification was based on published descriptions. Cultures of mycelia sterilia were subjected to molecular identification.

### Scanning electron microscopy (SEM)

SEM was used to observe ultrastructures of the sample and verify the observations made under the light microscope^39^. Fresh clean samples of the two types of wood were cut into 0.1 cm thick × 0.2 cm long segments and air-dried. Then the segments were coated with a 10 nm-thick gold film using a sputter coater. Morphological observations of the interactions between the fungi and their host were performed using a scanning electron microscope (SEM, JSM-6510LV, JEOL).

### DNA extraction, PCR amplification and sequencing

Endophytic fungi failing to sporulate were identified using molecular biological analysis of the internal transcribed region (ITS) of ribosomal DNA. The ITS region is relatively short (500–800 bp) and has been used for molecular characterisation of fungi. A large variety of primers are currently used for the amplification of different regions of fungal DNA^40^,^41^. A high quality and quantity of DNA is essential. After cultivation for 1 or 3 weeks at 25°C in the dark, all pure strains were subjected to DNA extraction, PCR amplification and sequencing. The partial nucleotide sequence of the internal transcribed spacer rDNA gene from an isolated endophytic fungus was amplified using the polymerase chain reaction (PCR) with the universal ITS primers ITS1 and ITS4^40^,^41^. DNA samples were amplified under the following reaction conditions: 3 min of initial denaturation at 94°C, 32 cycles of 30 s of denaturation at 94°C, 25 s of annealing at 55°C and 30 s of elongation at 72°C, 7 min of final elongation at 72°C. Single-band PCR products were purified using Watson's PCR purification kit (Watson, China). The purified PCR products were bidirectionally sequenced with an ABI3730 automated sequencer (Applied Biosystems, USA). All of the sequences obtained in this study were submitted to GenBank under the following accession numbers: KF019225, KF019226, KF019228, KF019229, KF019231, KF019232, KF019236, KF019237, KF019251, KF019252, KF019257, KF019282 and KF019253.

### Sequence analysis

The phylogenetic analysis of sequences was based on BLASTn searches of ITS sequence data in the GenBank database. A species was assigned when the identity between the experimental sequences and one in the database was greater than 99%, and the genus was assigned only when matches showed at least 95% identity. When the identity was less than 95%, the strain was considered unidentified. The determined ITS sequences were aligned to other sequences gained from the GenBank database with Clustal X 1.83, and then checked manually. Phylogenetic analysis was performed with MEGA 4.0^42^ using Kimura 2-parameter model with a transition to transversion ration^43^,^44^ to construct Neighbour-joining (NJ) trees.

### Statistical analysis

The colonisation frequency (CF%) of the endophyte species in the wood tissue was calculated as CF% = (Ncol/Nt) × 100, where Ncol represents the number of segments colonised by each endophyte and Nt the total number of segments studied^45^. The isolation frequencies (IF%) of fungal species were calculated as IF% = Ni/Nt × 100, where Ni represents the number of segments from which a given species were isolated and Nt the total number of segments used for isolation^46^.

## Author Contributions

S.S.S. and S.X.G. conceived and designed the experiments. S.S.S. performed the experiments. S.S.S. and X.Z. analyzed the data. S.S.S. and D.W.Z. wrote the manuscript. All authors reviewed the manuscript.

## Figures and Tables

**Table 1:** Endophyte isolation in two different *Dalbergia odorifera* T. Chen wood samples

wood types	segments examined	Total isolates	Endophytes species	Total CR (%)
purple-brown wounded wood	160	85	12	53.125
white healthy wood	160	2	1	1.25

**Table 2:** Isolation frequency (%) of endophytes in white healthy wood and purple-brown wound wood

white healthy wood	purple-brown wounded wood	Morphological identification	Fungal strain	GenBank accession No.
1.25%		*Bionectriaceae* sp.	12201	KF019253
	0.625%	*Fusarium* sp.1	12119	KF019225
	8.125%	*Phomopsis* sp.	12120	KF019226
	8.125%	*Oudemansiella* sp.	12122	KF019228
	21.25%	*Eutypa* sp.	12123	KF019229
	2.5%	*Fusarium* sp.2	12125	KF019231
	2.5%	*Eutypella* sp.1	12126	KF019232
	1.25%	*Fusarium* sp.3	12130	KF019236
	1.875%	*Fusarium* sp.4	12131	KF019237
	1.875%	*Auricularia polytricha*	12198	KF019251
	1.25%	*Eutypella* sp.2	12200	KF019252
	1.875%	*Pleosporales* sp.	12214	KF019257
	1.25%	*Exophiala jeanselmei*	12244	KF019282
